# Association between perinatal mood and anxiety disorder diagnosis and primary cesarean delivery among national Medicaid enrollees, 2017-2022

**DOI:** 10.3389/fpsyt.2026.1862736

**Published:** 2026-08-18

**Authors:** Gloria Sugg, Anca Tilea, Andrea Pangori, Stephanie V. Hall, Clayton J. Shuman, Kara Zivin, Vanessa Dalton, Karen M. Tabb

**Affiliations:** 1 School of Social Work, University of Illinois at Urbana-Champaign, Urbana, IL, United States; 2 Department of Obstetrics and Gynecology, University of Michigan, Ann Arbor, MI, United States; 3 Department of Psychiatry, University of Michigan Medical School, Ann Arbor, MI, United States; 4 Department of Systems, Populations and Leadership, School of Nursing, University of Michigan, Ann Arbor, MI, United States; 5 Program on Women’s Healthcare Effectiveness Research, Department of Obstetrics and Gynecology, University of Michigan Medical School, Ann Arbor, MI, United States; 6 Institute for Healthcare Policy and Innovation, University of Michigan, Ann Arbor, MI, United States; 7 Department of Health Policy and Management, University of Michigan School of Public Health, Ann Arbor MI, United States; 8 Center for Clinical Management Research, VA Ann Arbor Healthcare System, Ann Arbor, MI, United States

**Keywords:** cesarean delivery, perinatal anxiety, perinatal depression, perinatal mental health, perinatal mood and anxiety disorder (PMAD), women’s health

## Abstract

**Background:**

Cesarean deliveries can have an association with adverse maternal health outcomes, yet the relationship between perinatal mood and anxiety disorders (PMAD) and cesarean rates among publicly insured populations remains understudied and potentially underestimated.

**Materials and methods:**

Using the most current national Medicaid claims data from 2017 to 2022, this study examined the association between PMAD during pregnancy and primary cesarean delivery among reproductive-aged Medicaid beneficiaries with live births.

**Results:**

The study cohort included 3,365,248 Medicaid deliveries, of which 9.13% had a PMAD diagnosis during pregnancy. Adjusted logistic regression analyses revealed that women with PMAD had 13% greater odds of undergoing a primary cesarean delivery (Adjusted OR: 1.13, 95% CI: 1.12-1.14) than women without PMAD, after accounting for maternal age, race/ethnicity, metropolitan area residence status, and comorbid medical conditions.

**Conclusion:**

Among Medicaid enrollees, those with PMAD diagnoses had greater primary cesarean delivery rates than those without PMAD diagnoses. These findings highlight the complex interplay between maternal mental health and delivery outcomes. Study findings may support the development of effective healthcare strategies to address mental health and birth outcomes across diverse perinatal populations.

## Introduction

Perinatal mood and anxiety disorders (PMAD), which encompass depression and anxiety during pregnancy and postpartum, remain among the most common complications of childbearing in the United States (U.S.). Such perinatal mental health conditions affect approximately one in five women, and these conditions, including suicide and overdose, represent leading causes of pregnancy-related death. (1) As awareness of perinatal mental health has increased, national data demonstrate that PMAD prevalence among delivering women has risen substantially over time. (2) These trends underscore the importance of understanding how antenatal mental health conditions intersect with obstetric care and delivery outcomes.

Mental health conditions during pregnancy have an association with adverse perinatal outcomes, including preterm birth, low birth weight, and increased healthcare utilization. (3–5) Emerging evidence further suggests that women with PMAD may experience higher rates of cesarean delivery. For example, Zochowski et al. found that women with PMAD experienced significantly higher primary cesarean rates compared to those without PMAD in a commercially insured population. (6) Although often medically necessary, cesarean delivery involves longer recovery periods, greater clinical complexity, and can contribute to maternal emotional distress, (7) highlighting the importance of understanding to what extent antenatal mental health conditions affect delivery mode differences.

Public insurance finances more than 40% of U.S. births, yet much of the existing literature examining PMAD and delivery outcomes focuses on commercially insured cohorts. (7, 8) Mental health conditions and structural barriers including inadequate access to care also disproportionately affect publicly insured populations. (9) National inpatient data indicate that Medicaid covers a substantial proportion of deliveries complicated by serious mental illness and PMAD. (2) Despite high prevalence of PMAD and cesarean delivery, limited population-based evidence quantifies the association between PMAD diagnosed during pregnancy and primary cesarean delivery, defined as first cesarean in women with no prior cesarean history, among Medicaid populations.

Accordingly, this study examines the association between PMAD diagnosed during pregnancy and primary cesarean delivery among a national cohort of Medicaid enrollees from 2017 to 2022. By quantifying this association within a publicly insured population and adjusting for obstetric comorbidities and sociodemographic characteristics, we aimed to evaluate the relationship between antenatal PMAD and delivery mode across racial and ethnic groups.

## Materials and methods

This cohort study focused on women with live-birth deliveries from January 2017 through December 2022 using the national deidentified claims from Transformed Medicaid Statistical Information System (T-MSIS) Analytic Files (TAF). (10) This data source includes Medicaid-insured women across all 50 U.S. states and Washington D.C. and administrative records for inpatient and outpatient medical services, pharmacy prescriptions, and demographic characteristics. We identified deliveries using International Classification of Diseases, 10th Revision (ICD-10) diagnosis and procedure codes and Current Procedural Terminology (CPT) codes, following established methods for identifying obstetric deliveries. (11) Our cohort included women aged 15–44 with continuous enrollment for at least nine months prior to delivery and the month of the delivery. Because our unit of analysis is delivery, an individual may appear in the cohort more than once across the study period. We further restricted our cohort to those within states that had low or medium concern race and ethnicity data quality defined by Medicaid’s Data Quality Atlas. (12) States included in each year appear in Appendix Table A.

The outcome of interest was delivery type, categorized as primary cesarean section. We determined delivery type using ICD-10 and CPT procedure codes documented in Medicaid claims data. We identified individuals with a history of prior cesarean delivery using ICD-10 diagnosis codes and excluded these deliveries from the analytic cohort. The independent variable of interest was PMAD diagnosis during pregnancy. We identified PMAD as the presence of at least one inpatient or two outpatient claims in the nine months prior or the month of delivery using ICD-10 codes. Late December 2022 deliveries may not capture the full month of delivery since we do not have 2023 Medicaid claims. Code lists for delivery identification and PMAD diagnoses appear in the Appendix.

We included several covariates to account for potential confounding factors influencing cesarean delivery rates. We categorized maternal age 15–44 into sub-groups (15-19, 20-24, 25-30, 30-35, 36+), as prior research indicates that cesarean rates increase with maternal age. (3) We included race and ethnicity due to well-documented disparities in cesarean rates, with Black and Asian women experiencing higher cesarean rates compared to other groups. (13, 14) We incorporated metropolitan area versus non-metropolitan area residence status using Rural-Urban Commuting Area (RUCA) codes to account for geographic differences in healthcare access and provider practices, as prior research has shown higher cesarean rates in rural populations. (15) We included the Bateman Obstetric Comorbidity Index (OBCMI) to account for maternal health status when analyzing cesarean rates. This index provides a validated measure of maternal morbidity risk by incorporating preexisting conditions and pregnancy-related complications. (16) We categorized OBCMI as <2 (i.e., lower risk) or ≥2 (i.e., higher risk).

### Statistical analysis

We used descriptive statistics to summarize the study cohort’s demographic characteristics. We used logistic regression analysis to estimate the association between PMAD and primary cesarean delivery. The model adjusts for time, maternal age, race/ethnicity, metropolitan versus non-metropolitan residence status, and OBCMI. We also calculated the associated predicted probabilities of cesarean delivery to examine whether the relationship between PMAD and delivery varied by race and ethnicity. We used two-sided statistical tests with an alpha level of 0.05 for all statistical analyses. We conducted data management and statistical analyses using SAS version 9.4 (SAS Institute TM Cary, NC). The [Institution] Institutional Review Board approved this study as exempt due to the secondary analysis of deidentified data (Study Number).

## Results

**Table 1** summarizes the maternal characteristics of 3,365,248 Medicaid deliveries. Among them, 307,312 (9.13%) had a PMAD diagnosis during pregnancy. The overall age distribution reveals that most individuals were between 25–30 years old (35.16%), with similar patterns seen among those with (35.83%) and without PMAD (35.10%). The race and ethnicity distribution differed by PMAD status whereas those without PMAD reflected a similar distribution to the overall group. We identified that those with PMAD had a higher proportion of OBCMI ≥2 compared to those without PMAD (10.84% vs. 6.36%). Vaginal delivery remained the most common type of delivery overall and by PMAD status, but we found that those with PMAD had a slightly higher proportion of primary cesarean delivery (21.74%) compared to overall (19.68%) and without PMAD (19.47%).

**Table 1:** Maternal characteristics and cesarean delivery rates among medicaid beneficiaries overall and by perinatal mood and anxiety disorders (PMAD) status.

Maternal characteristics	Overall		With PMAD		Without PMAD	
	(N = 3,365,248)		(N = 307,312)		(N = 3,057,936)	
	n	(%)	n	(%)	n	(%)
Age						
15-19	298,977	8.88	24,624	8.01	274,353	8.97
20-24	988,754	29.38	84,755	27.58	903,999	29.56
25-30	1,183,314	35.16	110,101	35.83	1,073,213	35.10
31-35	595,195	17.69	58,619	19.07	536,576	17.55
36+	299,008	8.89	29,213	9.51	269,795	8.82
Race and ethnicity						
AIAN, non-Hispanic	56,568	1.68	5,935	1.93	50,633	1.66
Asian/Pacific Islander, non-Hispanic	85,895	2.55	2,868	0.93	83,027	2.72
Black, non-Hispanic	843,021	25.05	57,657	18.76	785,364	25.68
Hawaiian/Other Pacific Islander	20,596	0.61	1,300	0.42	19,296	0.63
Hispanic, all races	877,284	26.07	46,528	15.14	830,756	27.17
Multiracial, non-Hispanic	25,324	0.75	3,095	1.01	22,229	0.73
Other, non-Hispanic	108,472	3.22	8,917	2.90	99,555	3.26
White, non-Hispanic	1,296,213	38.52	176,863	57.55	1,119,350	36.60
Missing	51,875	1.54	4,149	1.35	47,726	1.56
Metropolitan status						
Metropolitan	2,732,008	81.18	237,624	77.32	2,494,384	81.57
Non-Metropolitan	612,972	18.21	67,677	22.02	545,295	17.83
Missing	20,268	0.60	2,011	0.65	18,257	0.60
OBCMI						
> 2	227,780	6.77	33,307	10.84	194,473	6.36
≤ 2	3,137,468	93.23	274,005	89.16	2,863,463	93.64
Delivery type						
Cesarean	662,211	19.68	66,807	21.74	595,404	19.47
Vaginal	2,703,037	80.32	240,505	78.26	2,462,532	80.53

This table presents demographic and clinical characteristics of Medicaid-enrolled individuals overall and among those diagnosed with perinatal mood and anxiety disorders (PMAD) during pregnancy and those not diagnosed. The dataset represents unique deliveries during the study period. AIAN, American Indian and Alaska Native.

We found that those with PMAD during pregnancy had 17% increased odds of primary cesarean delivery compared to those without PMAD when controlling for delivery year (Unadj OR: 1.17, 95% CI: 1.16-1.18) (Model not shown). When we additionally adjusted for age, race and ethnicity, OBCMI, and metropolitan residence status, we found that those with PMAD had 13% increased odds of primary cesarean delivery compared to those without PMAD (Adj OR: 1.13, 95% CI: 1.12 - 1.14, **Table 2**).

**Table 2:** Association between perinatal mood and anxiety disorders (PMAD) during pregnancy and primary cesarean deliveries.

Predictor	Odds Ratios	Lower 95% CI	Upper 95% CI
PMAD			
Yes vs No	1.13	**1.12**	**1.14**
**Year**	0.930	**0.929**	**0.932**
Age			
15–19 vs 20-24	0.96	**0.95**	**0.97**
25–30 vs 20-24	1.03	**1.02**	**1.04**
31–35 vs 20-24	1.22	**1.21**	**1.23**
36+ vs 20-24	1.42	**1.41**	**1.44**
Race/ethnicity			
AIAN, non-Hispanic vs White, non-Hispanic	0.81	**0.79**	**0.83**
Asian, non-Hispanic vs White, non-Hispanic	0.78	**0.76**	**0.79**
Black, non-Hispanic vs White, non-Hispanic	1.17	**1.16**	**1.18**
Hawaiian/Pacific Islander vs White, non-Hispanic	0.85	**0.82**	**0.88**
Hispanic, all races vs White, non-Hispanic	0.991	**0.984**	**0.999**
Multiracial, non-Hispanic vs White, non-Hispanic	0.963	**0.932**	**0.996**
Other, non-Hispanic vs White, non-Hispanic	1.04	**1.02**	**1.06**
OBCMI			
>2 vs (0,1)	1.97	**1.95**	**1.99**
Urbanicity			
Non-Metropolitan vs Metropolitan	1.04	**1.03**	**1.05**

*95% CI that hover around 1.00 are presented with 3 decimal places to identify statistical significance.

This table presents the adjusted model investigating the association between primary cesarean delivery and PMAD while adjusting for age, race/ethnicity, OBCMI, and metropolitan status. AIAN, American Indian and Alaska Native. Bold p-values indicate statistical significance at the alpha level of 0.05.

**Figure 1:**
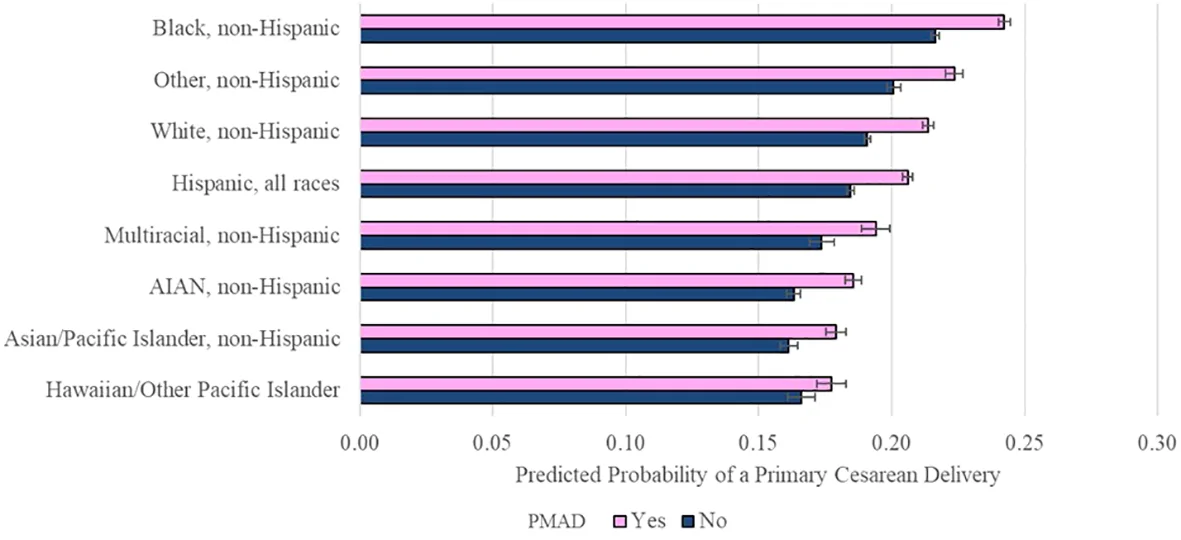
Predicted probability of cesarean delivery by race/ethnicity and perinatal mood and anxiety disorders (PMAD). This figure presents the predicted probability of cesarean delivery among Medicaid beneficiaries with and without perinatal mood and anxiety disorders (PMAD) by race/ethnicity group. Data are derived from adjusted logistic regression models that control for maternal age, race/ethnicity, the Obstetric Comorbidity Index (OBCMI), and metropolitan residence status.

**Figure 1** presents predicted probabilities of primary cesarean delivery by race and ethnicity and PMAD status. We found that across all race and ethnicity groups, those with PMAD had higher predicted probabilities of a primary cesarean delivery (18-24%) than those without (17-22%). Black non-Hispanic and Other, non-Hispanic had the highest predicted probabilities of a primary cesarean for both those with and without PMAD.

## Discussion

Our study found higher rates of primary cesarean delivery Medicaid beneficiaries with PMAD compared to those without PMAD. The findings indicate that PMAD diagnosis during pregnancy had a modest but statistically significant increased likelihood of primary cesarean delivery, even after adjusting for maternal age, race and ethnicity, obstetric comorbidity, and metropolitan area residence status (Adj OR: 1.13, 95% CI: 1.12–1.14). Predicted probabilities were highest among Black, non-Hispanic women in both PMAD groups; we present these descriptive patterns for context rather than as a separate inferential finding.

Prior research among commercially insured women found an approximately 3.5 percentage point higher predicted probability of primary cesarean delivery among those with PMAD. (6) The absolute differences observed for those with PMAD compared to those without PMAD in the present Medicaid cohort were 2.2% but directionally consistent. By extending this research to a national Medicaid population, the present study addresses an important evidence gap and demonstrates that this association exists within publicly insured populations.

Studies have also shown a rise in cesarean deliveries globally and within the United States (2, 17) among all populations. Dun and colleagues examined cesarean rates among three US states identifying low risk deliveries and finding race, ethnicity, private insurance, and private hospitals associated with higher rates of cesarean delivery. Globally the increase in cesarean can vary by availability of resources, preference, and a number of other factors.

Although modest, the observed effect size warrants interpretation. Unmeasured confounding related to the severity of mental health conditions, factors not captured in administrative claims data, could contribute to the observed effect (7). Accortt and colleagues (7) explored the association between PMADs and other comorbidities such as obesity whereas Wilson and colleagues (18) completed a systematic review and meta-analysis, finding an increased association between gestational diabetes and perinatal mental health conditions. Prior studies found that maternal mental health conditions, regardless of treatment status, have associations with adverse perinatal outcomes, highlighting importance of considering illness severity and access to care in evaluations of birth outcomes and modes of delivery. (19–21). The previous literature presented highlights the overall risk mothers with PMADs experience. Given the large sample size typical for Medicaid datasets, even small differences may reach statistical significance, further emphasizing the need for cautious interpretation.

Importantly, these findings raise questions about how maternal mental health during pregnancy may impact clinical risk factors or decision-making around delivery. Cesarean delivery itself has an increased risk of postpartum depression and post-traumatic stress symptoms, suggesting a potentially reinforcing cycle in which PMAD increase the likelihood of surgical delivery, which may in turn exacerbate postpartum mental health challenges. (1, 19) This bidirectional relationship highlights the need to better understand how mental health conditions shape obstetric care processes, including risk assessment, labor management, and thresholds for surgical intervention.

This study has some limitations. Claims data do not include information regarding the clinical indication for cesarean delivery, labor progression, patient preference, or provider decision-making; therefore, we cannot determine reasons underlying cesarean deliveries or whether PMAD diagnosis directly influenced delivery management. Although we adjusted for obstetric comorbidity using a validated index, residual confounding from unmeasured clinical or psychosocial factors may remain. The analysis relies on administrative claims data, which capture diagnoses and procedures for billing purposes rather than detailed clinical information. We identified PMAD status using diagnostic codes, which may underrepresent individuals with undiagnosed or undocumented mental health conditions. Because our study window included the delivery month, a small number of PMAD diagnoses recorded in the month of delivery may reflect early postpartum rather than antepartum encounters, potentially resulting in minor misclassification of PMAD timing. The data also do not provide information on symptom severity or timing of onset. Finally, because this study is observational, findings reflect associations and should not be interpreted as causal.

## Conclusion

Maternal mental health remains a critical public health issue, with depression and anxiety disorders associated with elevated risks of preterm birth, low birth weight, longer hospital stays, and increased healthcare utilization. (20–22) The use of a large, national Medicaid dataset represents a key strength of this study, enabling robust examination of cesarean delivery patterns across a racially and ethnically diverse population while minimizing recall. The significance of the results highlights the need for continued examination of PMADs impact on maternal health outcomes and the need for continued support for this population throughout pregnancy. Further research should examine how perinatal mental health treatment and interventions can reduce potential avoidable surgical deliveries. Increased access to structured maternal support programs such as collaborative care models which integrate behavioral health care into obstetrics care can positively influence mental health outcomes (23), and further studies could examine any links associated with mode of delivery. Additionally, qualitative research capturing the experiences of women managing PMADs during pregnancy may provide insight into how they navigate treatment decisions and birth planning, informing more patient-centered approaches to perinatal care. As maternal mental health remains a leading determinant of pregnancy-related outcomes, ensuring effective care for women with PMADs is essential.

## Data Availability

The original contributions presented in the study are included in the article/**Supplementary Material**. Further inquiries can be directed to the corresponding author.
