# Supplementary material for: Association between perinatal mood and anxiety disorder diagnosis and primary cesarean delivery among national Medicaid enrollees, 2017-2022
Source: Front Psychiatry. 2026 Aug 18;17:1862736. doi: 10.3389/fpsyt.2026.1862736 (PMC13531032; doi:10.3389/fpsyt.2026.1862736)
Supplement: Supplementary file 1 [file SupplementaryFile1.docx]

# **Appendix**

# **Delivery Codes**

### Cesarean delivery

| ICD-9-CM Px | 740, 741, 742, 744, 7499 |
| --- | --- |
| ICD-10-CM Px | 10D00Z0, 10D00Z1, 10D00Z2 |
| DRG | 370, 371, 765, 766, 540, 5401, 5402, 5403, 5404 |
| CPT | 59510, 59514, 59515, 59618, 59620, 59622 |

### Vaginal delivery

| ICD-9-CM Px |  |
| --- | --- |
| ICD-10-CM Px | 10D07Z3, 10D07Z4, 10D07Z5, 10D07Z6, 10D07Z7, 10D07Z8, 10E0XZZ |
| DRG | 372, 373, 374, 375, 767 768, 774, 775, 541, 542, 560, 5411, 5412, 5413, 5414, 5421, 5422, 5423, 5424, 5601, 5602, 5603 |
| CPT | 59400, 59409, 59410, 59610, 59612, 59614 |

# **Codes and process used to define PMAD**

**ICD-9 and ICD-10 codes used to identify maternal mental health disorders**

| Group | ICD Diagnosis Code |
| --- | --- |
| Anxiety | 300.xx, 308.xx, 313.xx, 293.xx, F06.xx, F40.xx, F41.xx, F42.xx, F43.xx, F48.xx, R45.xx |
| Depression | 311.xx, 296.xx, 300.xx, F32.xx, F33.xx |
|  |  |

**Codes and process used to define Bateman comorbidity**

Bateman BT, Mhyre JM, Hernandez-Diaz S, Huybrechts KF, Fischer MA, Creanga AA, Callaghan WM, Gagne JJ. Development of a comorbidity index for use in obstetric patients. Obstet Gynecol. 2013 Nov;122(5):957-965. doi: 10.1097/AOG.0b013e3182a603bb. PMID: 24104771; PMCID: PMC3829199.

| ICD 9 diagnosis codes | 291, 303, 3050,493, 394, 395, 396, 397, 424, 42822, 42823, 42832, 42833, 42842, 42843, 412, 413, 414, 581, 582, 583, 585, 587, 588, 6462, 7450, 7451, 7452, 7453, 7454, 7455, 7456, 7457, 7458, 7459, 7460, 7461, 7462, 7463, 7464, 7465, 7466, 7467, 7468, 7469, 7470, 7471, 7472, 7473, 7474, 6485, 304, 3052, 3053, 3054, 3055, 3056, 3057, 3058, 3059, 6483, 6423, 042, V08, 6424, 6427, 651, V272, 273, V274, V275, V276, V277, V278, 6410, 6411, 250, 6480, 401, 402, 403, 404, 405, 6420, 6421, 6422, 6427, 6542, 4160, 4168, 4169, 6425, 6466, 2824, 2826, 7100 |
| --- | --- |
| ICD 10 diagnosis codes | F10, J44, J45, I05, I06, I07, I08, I09, I34, I35, I36, I37, I38, I39, I500, I20, I25, N022, N03, N04, N05, N08, N171, N172, N18, N25, O268, Q20, Q21, Q22, Q23, Q24, Q25, Q26, O994, F11, F12, F13, F14, F15, F16, F18, F19, O13, O16, B20, B24, O987, Z21, O11, O14, O30, O31, Z372, Z373, Z374, Z375, Z376, Z377, Z3790, O44, E10, E11, O245, O246, O247, I10, I11, I12, I13, I15, O10, O11, O3420, I270, I272, I278, I279, O14, O15, D56, D57, M32 |

**Table A. States included by year based on race and ethnicity data quality**
